# Distinct Transcriptional Profile of PDZ Genes after Activation of Human Macrophages and Dendritic Cells

**DOI:** 10.3390/ijms23137010

**Published:** 2022-06-24

**Authors:** Jorge Rosas-García, Lucero A. Ramón-Luing, Karen Bobadilla, Marco Antonio Meraz-Ríos, Edgar E. Sevilla-Reyes, Teresa Santos-Mendoza

**Affiliations:** 1 Laboratory of Transcriptomics and Molecular Immunology, Instituto Nacional de Enfermedades Respiratorias Ismael Cosío Villegas, Mexico City 14080, Mexico; jorge.rosas@cinvestav.mx (J.R.-G.); karenbobadillalozoya@gmail.com (K.B.); 2 Department of Molecular Biomedicine, CINVESTAV, Mexico City 07360, Mexico; mmeraz@cinvestav.mx; 3 Laboratory of Integrative Immunology, Instituto Nacional de Enfermedades Respiratorias Ismael Cosío Villegas, Mexico City 14080, Mexico; ramonluing@yahoo.com.mx; 4 Centro de Investigación en Enfermedades Infecciosas, Instituto Nacional de Enfermedades Respiratorias Ismael Cosío Villegas, Mexico City 14080, Mexico

**Keywords:** PDZ, antigen-presenting cells, transcriptional signature, dendritic cells, macrophages, scaffold usage

## Abstract

The PDZ (PSD95, Dlg and ZO-1) genes encode proteins that primarily function as scaffolds of diverse signaling pathways. To date, 153 PDZ genes have been identified in the human genome, most of which have multiple protein isoforms widely studied in epithelial and neural cells. However, their expression and function in immune cells have been poorly studied. Herein, we aimed to assess the transcriptional profiles of 83 PDZ genes in human macrophages (Mɸ) and dendritic cells (DCs) and changes in their relative expression during cell PRR stimulation. Significantly distinct PDZ gene transcriptional profiles were identified under different stimulation conditions. Furthermore, a distinct PDZ gene transcriptional signature was found in Mɸ and DCs under the same phagocytic stimuli. Notably, more than 40 PDZ genes had significant changes in expression, with potentially relevant functions in antigen-presenting cells (APCs). Given that several PDZ proteins are targeted by viral products, our results support that many of these proteins might be viral targets in APCs as part of evasion mechanisms. Our results suggest a distinct requirement for PDZ scaffolds in Mɸ and DCs signaling pathways activation. More assessments on the functions of PDZ proteins in APCs and their role in immune evasion mechanisms are needed.

## 1. Introduction

Professional antigen-presenting cells (APCs), such as macrophages (Mɸ) and dendritic cells (DCs), are specialized cells involved in antigen uptake, processing, and presentation to initiate and regulate immune responses. These cells express pattern recognition receptors (PRRs) that detect pathogen-associated molecular patterns (PAMPs) produced by pathogens and damage-associated molecular patterns (DAMPs) produced by damaged host cells, activating APCs responses [1]. The mechanisms governing the activation of Mɸ and DCs after diverse stimuli have only been partially elucidated. The contribution and coordination of receptor-mediated signaling pathways and their downstream effector molecules remain unclear [2,3]. Scaffold proteins drive the spatial organization of cell signaling to guide the flow of molecular information that regulates cellular functions [4,5,6]. The PDZ (PSD-95/Dlg/ZO-1) domain is one of the most common interaction domains in scaffold proteins [7]. The primary function of proteins bearing such domains (PDZ proteins) is the assembly of protein complexes and their localization to specific regions within the cell, where they are involved in cellular processes such as cell–cell junctions, recycling, trafficking, and cell polarity [7,8]. PDZ proteins are found throughout different kingdoms, from bacteria to metazoan, highlighting their essential role in the coordination of signaling events [4,9]. This is reinforced by the fact that many PDZ proteins are subject of viral hijacking, such as the targeting of Scribble (Scrib) and Dlg1 by the NS1 protein of influenza A or PALS1 by the E protein of SARS-coronaviruses, which can result in immune evasion [9,10].

Recently, the relevant roles of some PDZ proteins have been demonstrated in APCs [11,12,13]. The scaffold function of the polarity protein, Scrib, is required for the correct assembly of the NADPH oxidase (NOX) complex in a PDZ domain-dependent manner and the generation of NOX-induced reactive oxygen species (ROS) in macrophages, which are indispensable for pathogen destruction [11]. In addition, Scrib and Dlg1 play important roles during antigen presentation as contributors to the expression of co-stimulatory molecules and IL-12 production during DC maturation, and can be targeted by influenza A virus [12,13]. Consequently, both proteins might be involved in the regulation of both innate and adaptive immune responses elicited by myeloid immune cells. 

PDZ proteins can be hypothesized to be widely used as platforms to orchestrate signaling events related to Mɸ and DC activation. Therefore, to gain further insights into the role of PDZ proteins in the activation of APCs, we assessed the transcriptional profile of 83 PDZ genes in Mɸ and DCs and evaluated the changes in their relative expression during cell stimulation with proinflammatory and phagocytic stimuli using high-throughput RT-qPCR. 

According to our findings, each cell type responds with distinct PDZ gene transcriptional signatures depending on the specific stimuli. After the same phagocytic stimulus, Mɸ and DCs triggered completely different PDZ expression profiles. Our results suggest a distinct requirement for PDZ scaffolds in APCs depending on the stimulus, thereby providing new insights into the role of scaffold proteins in APC function. Further, we identified several PDZ genes with potentially relevant functions in immune cells.

## 2. Results

### 2.1. Different PAMPs Induce Distinct PDZ Gene Expression Profiles in Human Monocyte-Derived Mɸ

**Figure 1:**
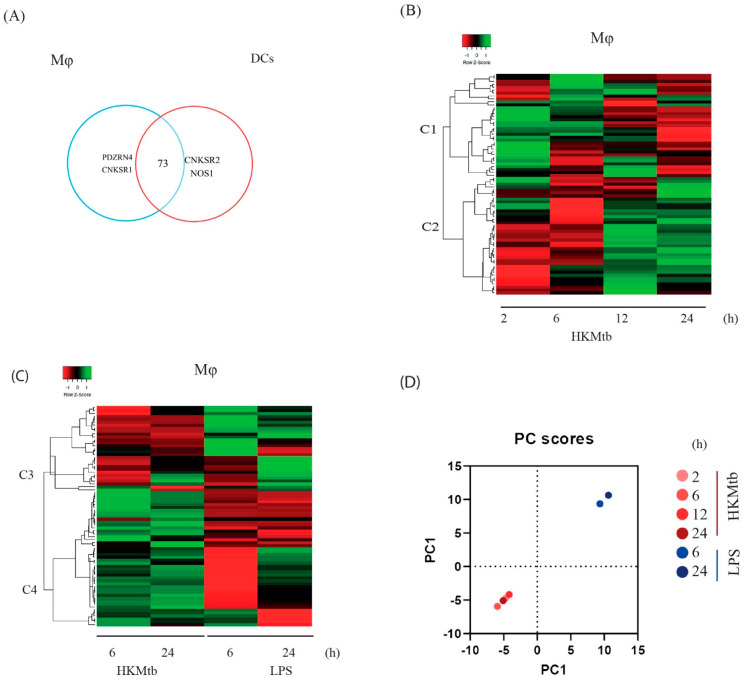
Different PAMPs induced specific PDZ gene expression patterns in human monocyte-derived Mɸ. (**A**) Venn diagram of PDZ genes expressed at baseline before stimulation in Mɸ and DCs. A total of 75 of the 83 gene panels were found to be expressed in Mɸ at baseline, eight genes were not expressed under basal conditions (*CARD10*, *CARD14*, *DLG2*, *MAGI1*, *MAGI2*, *MPP4, NOS1*, *CNKSR2*). (**B**) Heatmap showing hierarchical clustering of the PDZ gene expression profile changes of Mɸ stimulated with HKMtb referenced to unstimulated Mɸ. Data are presented as Z–score, corresponding to the change of expression of each time point respect to the mean of changes during the entire time course, where negative Z–score indicates a decrease (red), and positive Z–score indicates an increase (green) of each PDZ gene. (**C**) Heatmap showing hierarchical clustering of PDZ gene expression profile changes of Mɸ comparing HKMtb (left) or LPS (right) stimulation for 6 and 24 h, referenced to unstimulated Mɸ. Data are presented as Z–score, where negative Z–score indicates a decrease (red), and positive Z–score indicates an increase (green) gene expression. (**D**) PCA analysis of qPCR data from Mɸ stimulated with HKMtb at 2, 6, 12, 24 h and with LPS at 6 and 24 h. PC1 explains 81.50% of the variance; thus, it is the only component represented in the graphic.

We compared the changes in the relative expression of PDZ genes in Mɸ upon a phagocytic stimulus to that of a stimulus via TLR4. Mɸ were either exposed to heat-killed *Mycobacterium tuberculosis* H37Ra (HKMtb) for 2, 6, 12, and 24 h or stimulated with LPS for 6 and 24 h. *b2M*, *TBP*, and *GAPDH* were identified as the most stable reference genes for Mɸ under these stimulation conditions, (Supplementary Figure S1). The geometric mean of these genes was used to normalize changes in PDZ gene expression. Thereafter, the log2 fold-change values were transformed into Z-scores to compare the transcriptional profiles with both stimuli. A total of 75 of the 83 gene panels were found to be expressed in Mɸ and DC at baseline; two genes (*PDZRN4* and *CNKSR1*) were exclusively expressed by Mɸ (Figure 1A) and two other genes (*CNKSR2* and *NOS1*) were exclusively expressed by DC under basal conditions (Figure 1A). Although *NOS1* was not expressed under basal conditions in Mɸ, it was induced by stimuli. The overall Mɸ transcriptional response to HKMtb is summarized in a heatmap (Figure 1B). Using unsupervised hierarchical cluster analysis of the PDZ genes expressed at baseline and their changes with HKMtb stimulus at different time points, we identified two expression clusters (C) (C1 and C2) with a characteristic transcriptional response. We identified a biphasic reaction within both clusters: C1 contained PDZ genes that were mainly upregulated at 2 and/or 6 h and downregulated at 12 and 24 h. Instead, C2 had genes with decreased expression at 2 and 6 h but mainly increased at 12 and 24 h (Figure 1B). The PDZ genes with the most significant changes in gene expression (Z-ratio) after HKMtb stimulation are shown in Supplementary Table S1.

The transcriptional response of Mɸ stimulated with LPS was categorized into two gene clusters (Figure 1C). C3 grouped downregulated genes with HKMtb (at 6 and 24 h) that were mainly upregulated with LPS. In contrast, C4 genes were mainly upregulated by the phagocytic stimulus and downregulated by LPS. The set of PDZ genes with the most significant changes (Z-ratio) based on a comparison of both stimuli are shown in Supplementary Table S2. 

To visualize these changes globally, we applied the PCA method. The first principal component (PC1) explained 81.50% of the variance in the PDZ gene dataset, whereas PC2 explained 7.90%. According to clustering, the phagocytic stimuli (HKMtb) caused similar expression changes between time points, which grouped distinct from changes with LPS (Figure 1D).

### 2.2. Different PAMPs and DAMPs Induce Specific PDZ Gene Expression Patterns in Human Monocyte-Derived DCs

DCs were exposed to HKMtb or a maturation cocktail (MC) for 2, 6, 12, and 24 h to analyze the changes in the relative expression of PDZ genes. Under these stimulation conditions, we used *POLR2A*, *TBP*, and *GAPDH* as reference genes (Supplementary Figure S2). 

**Figure 2:**
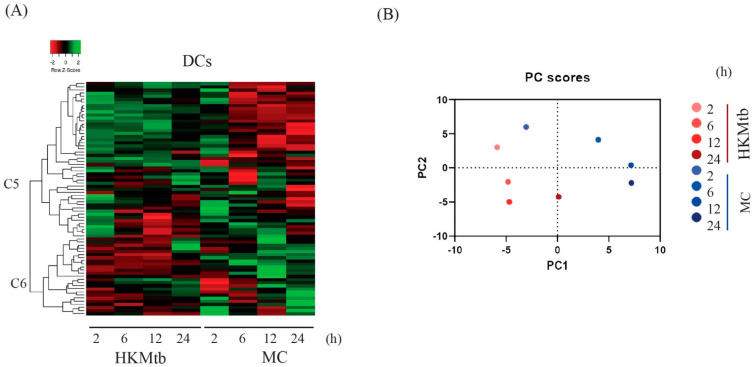
Different stimuli induced specific PDZ gene expression profiles in human monocyte-derived DCs. A total of 75 of the 83 gene panels were found to be expressed in DCs at baseline, eight genes were not expressed under basal conditions (*CARD10*, *CARD14*, *DLG2*, *MAGI1*, *MAGI2*, *MPP4*, *PDZRN4*, *CNKSR1*). (**A**) Heatmap showing hierarchical clustering of the PDZ gene expression profile changes of DCs at 2, 6, 12, and 24 h post–stimulation with HKMtb and MC, referenced to unstimulated DCs. Data are presented as Z–score, corresponding to the change of expression of each time point respect to the media of changes during the entire time course, where negative Z–score indicates a decrease (red), and positive Z–score indicates an increase (green) of each PDZ gene. (**B**) PCA analysis of qPCR data from DCs stimulated for 2, 6, 12, and 24 h with HKMtb or MC.

The overall transcriptional response of DCs to both stimuli is summarized in a heatmap (Figure 2A). We observed two main clusters, C5 and C6 (Figure 2A). According to the Z-score, C5 grouped mainly upregulated genes during the entire time course with HKMtb; however, with MC, these genes were upregulated at 2 h and downregulated from 6 h onwards. In contrast, PDZ genes in C6 were mainly downregulated during the HKMtb stimulation and were mainly upregulated with MC at 12 and 24 h. The PDZ genes with the most significant changes in gene expression (Z-ratio) are shown in Supplementary Table S3.

We proceeded to analyze the principal components of these data. PC1 explained 39.57% of the variance in the dataset, whereas PC2 explained 21.77%. Data showed pronounced differences during the entire time course with both stimuli, and dissimilar behavior between stimuli (Figure 2B).

### 2.3. HKMtb Phagocytosis Induces Distinct PDZ Gene Expression Profiles in Human Monocyte-Derived DCs and Mɸ

**Figure 3:**
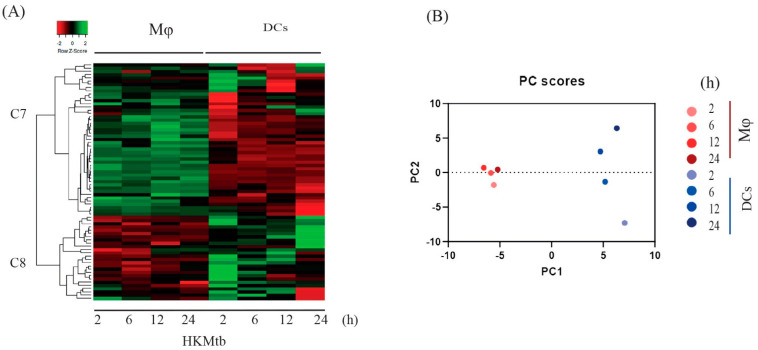
Phagocytosis of HKMtb induced distinct PDZ gene expression patterns in human monocyte-derived DCs and Mɸ. (**A**) Heatmap showing hierarchical clustering of the PDZ gene expression profile changes of both APCs stimulated at 2, 6, 12, and 24 h with HKMtb s referenced to unstimulated Mɸ and DCs. Data are presented as Z–score, corresponding to the change of expression of each time point respect to the mean of changes during the entire time course, where negative Z–score indicates a decrease (red), and positive Z–score indicates an increase (green) of each PDZ gene. (**B**) PCA analysis of qPCR data from Mɸ and DCs stimulated with HKMtb for 2, 6, 12, and 24 h.

We analyzed the transcriptional profiles of the 73 PDZ genes expressed at baseline (Figure 1A) in both APCs and those activated by HKMtb (Figure 3A); two main clusters were identified. C7 genes had a positive Z-score in Mɸ, while in DCs had a decreased expression during the stimulation time course. Conversely, in C8, PDZ gene expression was downregulated in Mɸ and upregulated in DCs during the entire time course. The most significant changes in gene expression (Z-ratio) are shown in Supplementary Table S4. 

Here, PC1 explained 53.74% of the variance in the data set, while PC2 explained 21.45% (Figure 3B), with a distinct behavior of Mɸ and DCs expression with the same phagocytic stimulus. The expression changes were subtle and similar between time points in Mɸ; however, in DCs, the changes were more pronounced and dissimilar between different time points (Figure 3B).

### 2.4. Differentially Expressed PDZ Genes (DEGs) Analysis

**Figure 4:**
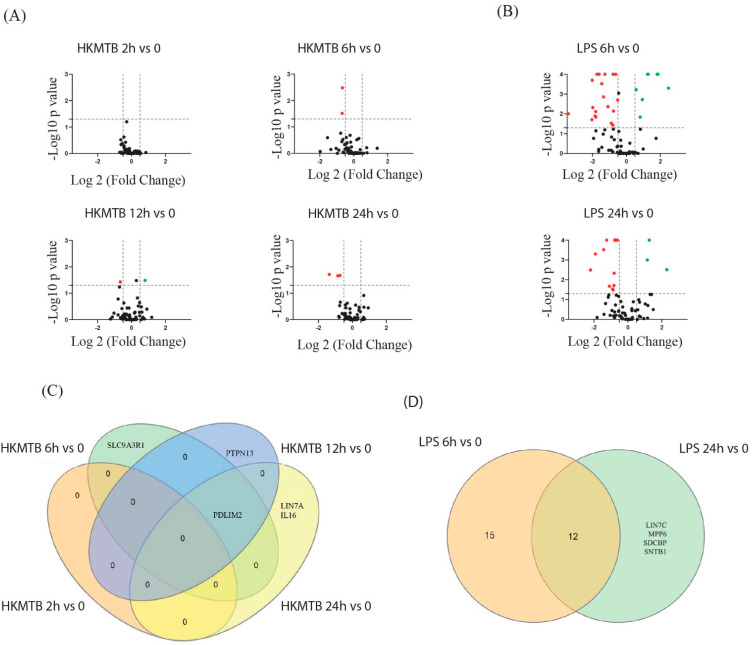
Differentially expressed PDZ genes in Mɸ stimulated with HKMtb and LPS at different time points. Volcano plots of DEGs in Mɸ at 2, 6, 12, and 24 h post–stimulation with HKMtb (**A**) and 6 and 24 h post–stimulation with LPS (**B**) compared to baseline expression in unstimulated Mɸ. Data are shown as the mean of log2 fold change subjected to Dunnett’s comparison (x–axis); log2 fold change ≥0.5 (green dots) or ≤–0.5 (red dots) are considered significant. The y–axis represents –log_10_ of the *p*–value; statistical significance was set at *p* < 0.05. Venn diagrams show DEGs comparison during time course stimulation with HKMtb (**C**) or LPS (**D**) in Mɸ. The set of DEGs is listed in Supplementary Table S5.

Data were expressed as log2 mean fold change from three to six healthy donors. The differentially expressed PDZ genes in Mɸ at 2, 6, 12, and 24 h post-stimulation with HKMtb (Figure 4A) and 6 and 24 h post-stimulation with LPS (Figure 4B) compared to baseline expression in unstimulated Mɸ (Figure 4) are shown. HKMtb stimulus induced a limited number of DEGs in Mɸ with zero genes at 2 h, two genes at 6 h, two genes at 12 h, and three genes at 24 h. Notably, PDLIM2 had significant changes from 6 h to 24 h with HKMtb stimulus (Figure 4C).

Notably, DEGs in Mɸ stimulated with LPS had more PDZ genes modified than HKMtb, with 27 genes at 6 h and 16 PDZ genes at 24 h, and a core of 12 genes changing from 6 to 24 h (Figure 4D and Supplementary Table S5). The set of DEGs for both stimuli in Mɸ is shown in Table 1.

**Figure 5:**
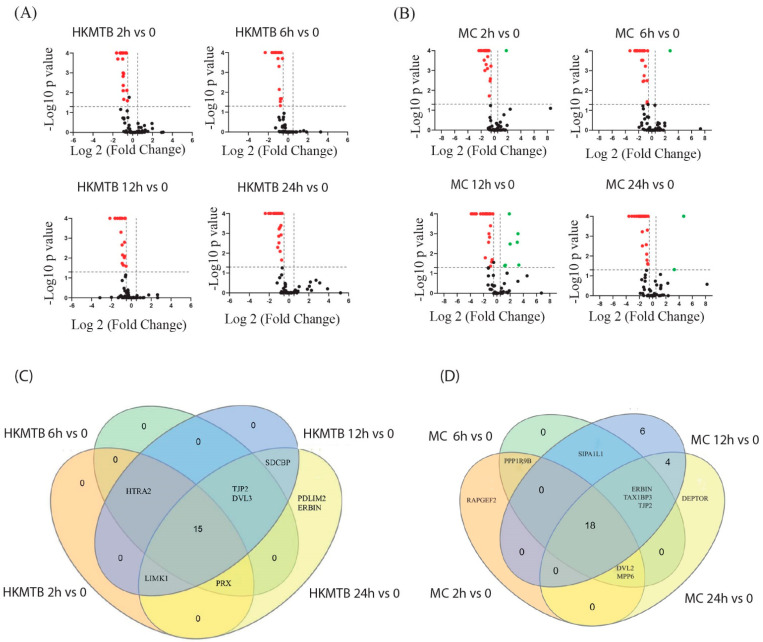
Differentially expressed PDZ genes in DCs stimulated with HKMtb and MC. Volcano plots of DEGs in DCs at 2, 6, 12, and 24 h post–stimulation with HKMtb (**A**) and MC (**B**) compared to baseline expression in unstimulated DCs. Data are shown as the mean of log2 fold change subjected to Dunnett’s comparison (x–axis); log2 fold change ≥0.5 (green dots) or ≤–0.5 (red dots) are considered significant. The y–axis represents –log_10_ of the *p*–value; statistical significance was set at *p* < 0.05. Venn diagrams show DEGs comparison during time course stimulation with HKMtb (**C**) (listed in Supplementary Table S6) or MC (**D**) (listed in Supplementary Table S7) in DCs.

DEGs in DCs at 2, 6, 12, and 24 h post-stimulation with HKMtb (A) and MC (B) are shown in Figure 5 and listed in Table 2. HKMtb induced 18 DEGs at 2 h, 19 at 6 h, 20 at 12 h, and 22 genes at 24 h (Figure 5C and Table 2). All DEGs were downregulated for all stimulation times. A core of 15 of these DEGs was modified from 2 to 24 h (Supplementary Table S6).

DEGs in DCs with MC stimulation are shown in Figure 5B. Twenty-one genes were downregulated, while only one was upregulated at 2 h; 24 genes were downregulated and only one was upregulated at 6 h; 25 genes were downregulated and seven were upregulated at 12 h; and 26 genes were downregulated and two were upregulated at 24 h (Table 2). A core of 18 DEGs was modified from 2 h to 24 h (Figure 5D and Supplementary Table S7).

**Figure 6:**
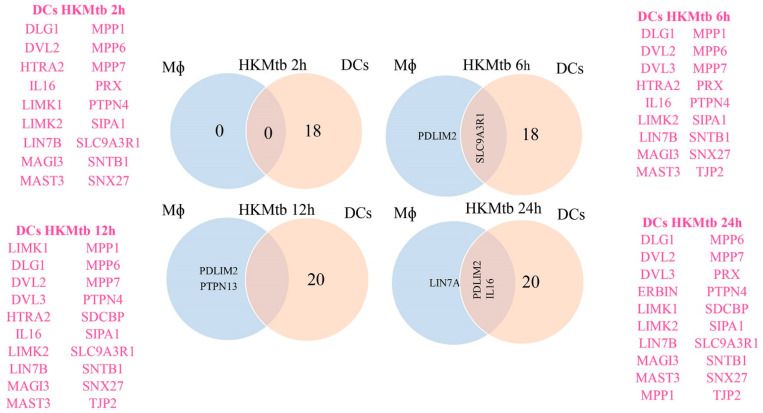
Differential expression of PDZ genes upon stimulation with HKMtb in Mɸ and DC. Venn diagram showing DEGs sharing by both APCs at specific time points during the entire time course stimulation by HKMtb.

We proceeded to compare the DEGs of Mɸ and DCs exposed to HKMtb, as shown in Figure 6. Mɸ and DCs showed pronounced differences in PDZ gene regulation during the phagocytosis of HKMtb, where DEGs were mainly found in DCs.

## 3. Discussion

Scaffold proteins are of the highest relevance in the organization of precise cellular responses to specific stimuli. PDZ proteins can improve cellular fitness by forming transient complexes that support several cellular processes, such as proliferation, apoptosis, stress adaptation, stemness, and organelle biology [14]. There are several examples illustrating how dysfunctions in PDZ proteins or their targeting by viral proteins alter cellular fitness functions and may be involved in mechanisms for immune evasion by viruses [9,10,15,16,17,18].

Recently, in addition to our group, other researchers gained an insight into Scrib and Dlg1, which are two PDZ proteins governing apicobasal epithelial cell polarity (ABCP) in the central signaling pathways of Mɸ and DCs in mice and humans [11,12,13,19]. Scrib and Dlg1 are expressed and regulated in human APCs, including monocytes, Mɸ, and DCs, and play key roles in the expression of co-stimulatory molecules and IL-12 production in DCs where both proteins can be targeted by Inlfuenza A virus [12,13]. Owing to this knowledge and the evolutionary conservation of these scaffolding proteins, we hypothesized that more members would support several cellular processes involved in the activation of APCs. 

Here, large-scale PDZ gene expression analysis was performed in Mɸ and DCs stimulated via diverse PRRs. We analyzed the transcriptional profiles of 83 PDZ genes in Mɸ and DCs by RT-qPCR. Most of the genes (90%) were found to be expressed at baseline in both Mɸ and DCs (Figure 1A). Further, 41.3% of these genes significantly modified their transcriptional profile after LPS stimulation, while only 6.7% changed after phagocytosis in Mɸ (Figure 4). For DCs, 30.6% of the basally expressed PDZ genes were significantly changed upon phagocytosis while 49.3% were changed upon DC stimulation with a cocktail of inflammatory cytokines (Figure 5). Therefore, inflammatory stimuli, such as LPS and MC, induced more significant transcriptional modification of PDZ genes than phagocytosis of HKMtb (Figure 4). Moreover, HKMtb caused larger transcriptional changes in DCs than Mɸ (Figure 6); this finding correlates with previous reports, demonstrating that DCs are more responsive than Mɸ following the exposure to different types of stimuli [20,21]. 

We previously studied the functions of Dlg1 and Scrib in human DCs. Here, we demonstrated that the mRNA of Dlg1 changed abruptly and significantly in LPS-stimulated Mɸ (Figure 4 and Supplementary Table S5). Further, the mRNA levels of Dlg1 decreased substantially with both stimuli used in DCs during the entire time course (Figure 5, Supplementary Figures S6 and S7). Owing to our extensive analysis of PDZ genes, we identified additional PDZ genes with significant transcriptional changes in Mɸ and DCs under different stimulation conditions (Table 1 and Table 2). Our findings suggest that these PDZ proteins have relevant functions in APC signaling pathways.

ZO-1 (TJP-1) and ZO-2 (TJP-2) are PDZ proteins commonly related to ABCP and the maintenance of tight junctions (TJ). In response to proinflammatory cytokines or bacterial products, such as LPS, the expression of ZO-1 is reduced and redistributed away from the TJ, resulting in increased epithelial permeability [22,23,24]. However, in the immune system, LPS can trigger the reorganization of TJ proteins with the upregulation of ZO-1 in mouse DCs. This ZO-1 increment appears to be related to the emission mechanism of projections across the epithelial mucosa to sample the exterior environment or a possible role in regulating gap junction intercellular communication between immune cells and epithelial cells [25,26]. Consistently, we found that ZO-1 mRNA levels increased in Mɸ stimulated with LPS. Such behavior may be related to the regulation of the hetero-cellular interaction of Mɸ with epithelial cells [26]. In addition, ZO-1 mRNA was basally expressed in human DCs and abruptly increased at 2 h after a phagocytic stimulus (Supplementary Table S3); however, this change was not significant. We did not stimulate DCs with LPS. Further, we cannot exclude the possibility that this and other stimuli may induce considerable increases in ZO-1 mRNA levels. 

ZO-2 is involved in the molecular organization at the immune synapse (IS) in T cells, mediating the formation of a type of gap junction through interaction with connexin 43 [27]. Whether or not ZO-2 also regulates IS organization on the APC side, as demonstrated for Scrib, would be an interesting finding [12]. ZO-2 downregulation has been shown to result in increased STAT1 expression and transcriptional activity [28]. Further, STAT1 activity is known to promote DC maturation [29,30]. ZO-2 mRNA expression was found to significantly reduce in DCs with both stimuli (Figure 5, Table 2), which may correlate with the maturation process elicited by stimulation.

*PDLIM2* had significant changes during the entire time course of stimulation with all stimuli both in Mɸ and DCs (Figure 4 and Figure 5 and Supplementary Tables S5 and S7). PDLIM2 is expressed in several immune cells and is a central negative regulator of NF-κB transcription factor activity, thereby downregulating inflammatory cytokine production [31,32]. PDLIM2 can shuttle between the nucleus and cytoplasm, and additional functions have been described for cytoplasmic PDLIM2, including adhesion promotion [33]. In Mɸ, both stimuli caused a significant decrease in PDLIM2, with LPS having a greater significant effect. In DCs, stimulation with MC, which simulates an inflammatory environment, elicited the most significant decrease in PDLIM2 during the entire time course. Our results suggest that changes in the mRNA expression of PDLIM2 contribute to the regulation of its functions in Mɸ and DCs stimulated through diverse PRRs.

*IL-16* decreased the mRNA levels in both Mɸ and DCs with all stimuli tested (Figure 4 and Figure 5 and Supplementary Tables S5–S7). The mRNA levels of IL-16 decreased in T cells upon activation [34,35]. IL-16 was initially reported as a T cell chemoattracting cytokine [36] and is known as an immunomodulator that impairs antigen-induced T-cell activation [37,38,39]. Identifying the immunomodulatory function of IL-16 in Mɸ and DCs would serve as a meaningful finding. 

According to our experimental strategy, HKMtb stimulation favors type II phagocytosis, mainly regulated by complement receptor 3 (CR3). Functional outcomes after phagocytosis are different between Mɸ and DCs. Although Mɸ efficiently destroy phagocytosed pathogens, DCs display limited capacity for lysosomal degradation for the preservation of peptides for their primary function of antigen processing for presentation to T lymphocytes [40]. Mɸ phagosomes progressively acquire the lysosomal degradative enzymes during their maturation into phagolysosomes [41]. DC phagolysosome maturation instead, contains fewer degradative enzymes and recruits protease inhibitors, leading to a controlled degradative milieu [42,43]. Differences between Mɸ and DCs in terms of their proteolytic system and pH control during phagolysosome maturation [44,45] suggest different components and temporality forming specific protein complexes therefore, distinct use of scaffolds. We identified a distinct transcriptional signature after the phagocytosis of HKMtb into Mɸ versus DCs (Figure 3). Of note, only five PDZ DEGs were found in Mɸ (Figure 4), whereas a core of 15 PDZ DEGs was found to be downregulated in DCs during the entire time course; some additional PDZ DEGs were found to only change at specific time points (Figure 5). Therefore, the particular characteristics of Mɸ and DCs related to the outcome of each cell after phagocytosis may be supported by specific PDZ scaffold proteins.

In previous assessments using a similar experimental model (human monocyte-derived Mɸ and DCs), the existence of cell- and stimulus-specific responses in both cell types for immune effector molecules, such as cytokines and chemokines, was revealed [20]. Our results suggest that these specific signaling pathways require organization by specific PDZ scaffolding proteins.

The abovementioned PDZ genes have important functions in immune cells. Our analysis of 83 PDZ genes in only two immune cell types subjected to few stimuli revealed the significant transcriptional changes during the activation of Mɸ and DCs through diverse PRRs (Figure 4 and Figure 5). To date, 153 PDZ genes have been identified in the human genome (Supplementary Table S8); thus, more research is needed in this emerging field to assess other PDZ genes in APCs that are not included in this study. Further, the network of PDZ scaffolds involved in responses to other PRRs in additional immune cells, such as lymphocytes, neutrophils, or mast cells, should be elucidated.

In conclusion, our results highlight a distinct signature of PDZ gene expression in Mɸ and DCs related to their specialized functions after stimulation with diverse PRRs, suggesting a distinct use of this type of scaffolds. Further analyses are required to clarify the impact of each PDZ protein scaffold on the functions of Mɸ and DCs as well as their potential in immune evasion mechanisms elicited by viral pathogens such as influenza A, SARS-coronaviruses or adenoviruses.

Our findings contribute to a better understanding of the role of PDZ scaffold proteins in the general signaling pathways of APC activation and may lead to establish the foundation for exploring the functions of PDZ proteins not only in Mɸ and DCs but also in other immune cells.

## 4. Materials and Methods

### 4.1. Ethics Statement 

Buffy coats were obtained from healthy human donors that visited the Blood Bank at the Instituto Nacional de Enfermedades Respiratorias, Mexico City, Mexico, after approval by the institutional scientific and ethics committees (IRB# B16-20 approved in 2020). This study was conducted in accordance with the principles of the declaration of Helsinki. Written informed consent was not required following national legislation and institutional requirements.

### 4.2. Mɸ and DC Differentiation and Stimulation Conditions

Peripheral blood mononuclear cells (PBMCs) were isolated from buffy coats using a Lymphoprep^TM^ (Alere Technologies, Oslo, Norway) density gradient. CD14+ cells were purified using human antibody-CD14-labeled magnetic microbeads and LS columns (Miltenyi Biotec, Auburn, CA, USA). CD14+ cells were seeded in RPMI 1640 medium (Corning, New York, NY, USA) supplemented with 10% fetal calf serum (HyClone, New Hampshire, UT, USA), 100 U/mL penicillin, 100 μg/mL streptomycin, and 200 mM l-glutamine (Sigma-Aldrich, St. Louis, MO, USA). As previously described [12], monocytes were cultured with GM-CSF (10 ng/mL) (R&D System, Minneapolis, MN, USA) to obtain Mɸ, and with GM-CSF (53 ng/mL) and IL-4 (25 ng/mL) (R&D System, Minneapolis, MN, USA) to obtain DCs.

Mɸ were stimulated with LPS (100 ng/mL) for 6 and 24 h and DCs with a maturation cocktail (MC) (10 ng/mL TNF-α, 10 ng/mL IL-1β, and 1 μg/mL PGE2 (Biolegend, San Diego, CA, USA) for 2, 6, 12, and 24 h. Mɸ and DCs were also stimulated with heat-killed *Mycobacterium tuberculosis* H37Ra strain (HKMtb) at a multiplicity of infection (MOI) of 1:10 (1 cell per 10 bacilli) for 2, 6, 12, and 24 h; HKMtb were previously incubated in a medium containing 10% FCS at 37 °C to favor type II phagocytosis.

### 4.3. RNA Purification, cDNA Synthesis, and Pre-Amplification

Total RNA was extracted using a Quick-RNA MiniPrep kit (Zymo Research, Irvine, CA, USA) and quantified using the Qubit™ assay kit and a Qubit 2.0 Fluorometer (Life Technologies, Waltham, MA, USA).

For expression analysis, 64 PDZ genes were selected from PDZ genes with high and medium expression levels in diverse immune cells as previously reported [46]. The gene set was completed with 11 PDZ genes belonging to the MAGUK subfamily and 8 PDZ genes involved in trafficking, signaling, and recycling processes. Five reference genes were included in the relative expression analyses. Primers were designed using a Deltagene assay (Fluidigm, South San Francisco, CA, USA) and are listed in Supplementary Table S9.

Total RNA (200 ng of total RNA in a 20 μL volume reaction) was used to synthesize cDNA using the OneStep RT-PCR Kit (Qiagen, Hilden, Germany). A pool of the 88 primer pairs (Supplementary Table S9) was used under the following conditions: 50 °C for 30 min, 95 °C for 15 min, followed by 15 cycles of pre-PCR cycling amplification at 94 °C for 30 s, 60 °C for 60 s, and 72 °C for 60 s. The pre-amplified product was diluted 15-fold with Tris/Ethylenediaminetetraacetic acid (TE) Buffer.

### 4.4. Real-Time PCR

qPCR was performed on a BioMark 48.48 Integrated Fluidic Circuit (IFC) (Fluidigm, South San Francisco, USA) combining 48 pre-amplified samples with 48 pairs of primers for multi-parallel qPCR reactions. The sample mix included: SSoFast Master Mix (Bio-Rad, Hercules, CA, USA) and 20× sample loading reagent (Fluidigm, South San Francisco, CA, USA) for each pre-amplified cDNA. Each of the 88 assay mixes was prepared with 100 μM of the respective primer pair and DNA suspension buffer (Buffer TE). Thermal cycling and detection were conducted using a BioMark instrument (Fluidigm, South San Francisco, CA, USA) with the following conditions: 15 min at 95 °C, 30 cycles at 94 °C for 10 s, 54 °C for 30 s, and 72 °C for 10 s. Melt curve analysis (70–90 °C) was performed following PCR amplification to assess each amplicon. Cq values and amplification curves were analyzed using Real-Time PCR Analysis software 4.5.2 (Fluidigm, South San Francisco, CA, USA).

### 4.5. Data Analysis

The qBase  +  software package (BioGazelle, Zwijnaarde, Belgium, https://www.qbaseplus.com/ accessed on 15 February 2021) was used to determine the reference gene expression stability with the application of the geNorm algorithm [47] to identify the most stable reference genes (geNorm M) and the pairwise variation using n or n  +  1 reference genes to determine the lowest number of reference genes required for accurate normalization (geNorm V), according to the MIQE guidelines [47,48]. The relative expression of each target gene was determined using three to six independent cell donor samples (biological replicates) and three technical replicates per sample and compared to the chosen references. We normalized raw qPCR data (cycle quantification values (Cq)) with the geometric mean value of the most stable reference genes for each cell type (*GAPDH*, *TBP* and *b2M* genes were the most stable for Mɸ, while *GAPDH*, *TBP* and POLR2A for DCs). We calculated the relative expression of each PDZ gene in each condition compared to the non-stimulated condition and built a fold-change matrix. This matrix was used to Z transformation and DEGs analyses.

For differentially expressed genes (DEGs), data are expressed as log2 mean fold change. Normality was assessed using the Kolmogorov–Smirnov test. One-way ANOVA with Dunnett’s test was used to compare the expression following each treatment to the baseline expression in unstimulated Mɸ or DCs. Statistical significance was set at *p* < 0.05. Non-supervised global gene expression analysis was performed using principal component analysis (PCA). DEG analyses and PCA were performed using GraphPad Prism version 9.1.2 (GraphPad Software, San Diego, CA, USA). 

The RT-qPCR data were transformed to Z scores and clustered using the average linkage method with the Pearson correlation distance metric by the Heatmapper webserver (http://www.heatmapper.ca/expression/ accessed on 11 April 2022). Then, Z scores were used to calculate Z-ratio. A Z-ratio ˃ 2.0 or < –2.0 was considered significant for highlighting conditions in which a particular gene had peak changes in gene expression in the Z-ratio during the time course of its stimulation [49]. Venn diagrams were constructed using the InteractiVenn web server (http://www.interactivenn.net/ accessed on 19 April 2022).

## Figures and Tables

**Table 1:** PDZ DEGs in Mɸ stimulated with HKMtb or LPS. Gene expression level (log2 mean fold change) subjected to one-way ANOVA with a Dunnett’s test. Statistical significance was set at *p* < 0.05.

Mɸ											
HKMtb								LPS			
HKMtb 2 h vs. 0		HKMtb 6 h vs. 0		HKMtb 12 h vs. 0		HKMtb 24 h vs. 0		LPS 6 h vs. 0		LPS 24 h vs. 0	
Gene Name	Log2 (FC)	Gene Name	Log2 (FC)	Gene Name	Log2 (FC)	Gene Name	Log2 (FC)	Gene Name	Log2 (FC)	Gene Name	Log2 (FC)
		SLC9A3R1	−0.67	PTPN13	0.80	IL16	−1.36	ARHGEF11	1.25	LIMK2	2.30
		PDLIM2	−0.69	PDLIM2	−0.66	PDLIM2	−0.73	CYTIP	0.81	SIPA1L1	1.26
						LIN7A	−0.86	DVL3	0.57	TJP1	1.14
								LIMK2	2.48	DLG1	−0.78
								RAPGEF2	1.82	HTRA1	−0.82
								SIPA1L1	1.28	IL16	−2.21
								TJP1	1.86	LIN7A	−1.92
								TJP2	0.94	LIN7C	−0.62
								CARD11	−1.85	MPP6	−0.90
								DLG1	−0.79	PDLIM2	−1.43
								DVL2	−1.99	RAPGEF6	−0.81
								HTRA1	−0.79	RGS12	−0.77
								IL16	−1.81	SDCBP	−0.72
								LIN7A	−2.02	SLC9A3R1	−1.25
								MAST2	−1.09	SNTB1	−1.09
								MAST3	−1.81	TIAM1	−0.88
								MPP5	−0.64		
								PDLIM2	−1.45		
								PPP1R9B	−1.27		
								PTPN13	−3.43		
								PTPN4	−1.35		
								RAPGEF6	−0.76		
								RGS12	−0.52		
								SIPA1L3	−1.61		
								SLC9A3R1	−1.74		
								SLC9A3R2	−2.04		
								TIAM1	−0.90		

**Table 2:** PDZ DEGs in DCs stimulated with HKMtb and MC. Gene expression level (log2 mean fold change) subjected to one-way ANOVA with a Dunnett’s test. Statistical significance was set at *p* < 0.05.

DCs															
HKMtb								MC							
HKMtb 2 h vs. 0		HKMtb 6 h vs. 0		HKMtb 12 h vs. 0		HKMtb 24 h vs. 0		MC 2 h vs. 0		MC 6 h vs. 0		MC 12 h vs. 0		MC 24 h vs. 0	
Gene Name	Log2(FC)	Gene Name	Log2(FC)	Gene Name	Log2(FC)	Gene Name	Log2(FC)	Gene Name	Log2(FC)	Gene Name	Log2(FC)	Gene Name	Log2(FC)	Gene Name	Log2(FC)
DLG1	−0.74	DLG1	−0.69	DLG1	−0.66	DLG1	−0.84	RAPGEF2	1.80	SIPA1L1	2.76	APBA3	2.00	DEPTOR	4.69
DVL2	−0.95	DVL2	−0.88	DVL2	−0.89	DVL2	−0.74	DLG1	−1.01	DLG1	−1.13	CYTIP	3.09	PATJ	3.28
HTRA2	−0.96	DVL3	−0.56	DVL3	−0.61	DVL3	−0.70	DVL2	−1.24	DVL2	−1.16	PATJ	3.32	ARHGAP21	−0.74
IL16	−1.62	HTRA2	−0.77	HTRA2	−0.81	ERBIN	−1.03	DVL3	−0.51	DVL3	−0.64	PPP1R9A	3.19	DLG1	−1.07
LIMK1	−0.53	IL16	−1.22	IL16	−1.64	IL16	−2.39	HTRA2	−1.53	ERBIN	−1.37	PTPN13	1.29	DVL2	−0.72
LIMK2	−1.16	LIMK2	−1.22	LIMK1	−0.53	LIMK1	−0.79	IL16	−2.06	HTRA2	−1.19	SIPA1L1	1.88	DVL3	−0.68
LIN7B	−0.90	LIN7B	−0.78	LIMK2	−1.17	LIMK2	−1.15	LIMK1	−1.26	IL16	−3.31	TIAM1	1.19	ERBIN	−1.69
MAGI3	−1.01	MAGI3	−1.01	LIN7B	−0.93	LIN7B	−1.14	LIMK2	−2.34	LIMK1	−2.28	ARHGAP21	−1.09	HTRA2	−0.86
MAST3	−0.91	MAST3	−0.91	MAGI3	−1.06	MAGI3	−0.91	LIN7B	−1.43	LIMK2	−1.86	DLG1	−1.13	IL16	−3.58
MPP1	−1.02	MPP1	−1.24	MAST3	−0.66	MAST3	−1.07	MAGI3	−1.25	LIN7B	−1.32	DVL3	−0.60	LIMK1	−2.19
MPP6	−0.58	MPP6	−0.99	MPP1	−1.62	MPP1	−2.03	MAST3	−1.01	MAGI3	−0.98	ERBIN	−1.63	LIMK2	−1.91
MPP7	−1.24	MPP7	−2.31	MPP6	−0.92	MPP6	−0.98	MPP1	−0.59	MAST3	−1.35	HTRA2	−0.79	LIN7B	−1.57
PRX	−1.50	PRX	−0.77	MPP7	−2.16	MPP7	−1.85	MPP6	−0.65	MPP1	−2.55	IL16	−3.63	MAGI3	−1.69
PTPN4	−1.26	PTPN4	−1.20	PTPN4	−1.15	PDLIM2	−1.01	MPP7	−1.46	MPP6	−0.70	LIMK1	−2.47	MAST2	−1.54
SIPA1	−0.97	SIPA1	−0.90	SDCBP	−0.70	PRX	−0.90	PDLIM2	−1.61	MPP7	−2.56	LIMK2	−1.83	MAST3	−1.73
SLC9A3R1	−0.96	SLC9A3R1	−1.42	SIPA1	−0.69	PTPN4	−1.38	PPP1R9B	−0.76	PDLIM2	−2.14	LIN7B	−1.25	MPP1	−3.15
SNTB1	−0.96	SNTB1	−1.61	SLC9A3R1	−1.04	SDCBP	−0.99	PRX	−1.93	PPP1R9B	−0.72	MAGI3	−0.95	MPP6	−0.80
SNX27	−0.64	SNX27	−0.72	SNTB1	−1.39	SIPA1	−0.81	PTPN4	−1.16	PRX	−1.53	MAST2	−0.96	MPP7	−1.97
		TJP2	−0.68	SNX27	−0.63	SLC9A3R1	−1.56	SIPA1	−0.96	PTPN4	−1.77	MAST3	−1.46	PDLIM2	−2.78
				TJP2	−0.93	SNTB1	−1.28	SLC9A3R1	−1.37	SIPA1	−1.47	MPP1	−3.87	PRX	−0.90
						SNX27	−0.80	SNTB1	−1.05	SLC9A3R1	−2.43	MPP7	−1.68	PTPN4	−1.18
						TJP2	−1.46	SNX27	−0.81	SNTB1	−2.33	PDLIM2	−3.32	SDCBP	−1.37
										SNX27	−1.11	PRX	−1.20	SIPA1	−0.96
										TAX1BP3	−0.70	PTPN4	−1.46	SLC9A3R1	−2.00
										TJP2	−2.27	SDCBP	−1.17	SNTB1	−2.45
												SIPA1	−1.62	SNX27	−1.28

## Data Availability

The raw data supporting the conclusions of this article will be made available by the authors, without undue reservation.

## Supplementary Materials

The following supporting information can be downloaded at: https://www.mdpi.com/article/10.3390/ijms23137010/s1.
