# Supplementary material for: Distinct Transcriptional Profile of PDZ Genes after Activation of Human Macrophages and Dendritic Cells
Source: Int J Mol Sci. 2022 Jun 24;23(13):7010. doi: 10.3390/ijms23137010 (PMC9266728; doi:10.3390/ijms23137010)
Supplement: Supplementary file 1 [file ijms-23-07010-s001.zip › ijms-1755072-supplementary.pdf]

Supplementary Material

Supplementary Figure S1. geNorm M and V analysis in Mϕ. Before data and statistical analyses, the most stable reference genes for Mϕ under these stimulation conditions were calculated according to geNorm M and geNorm V values.

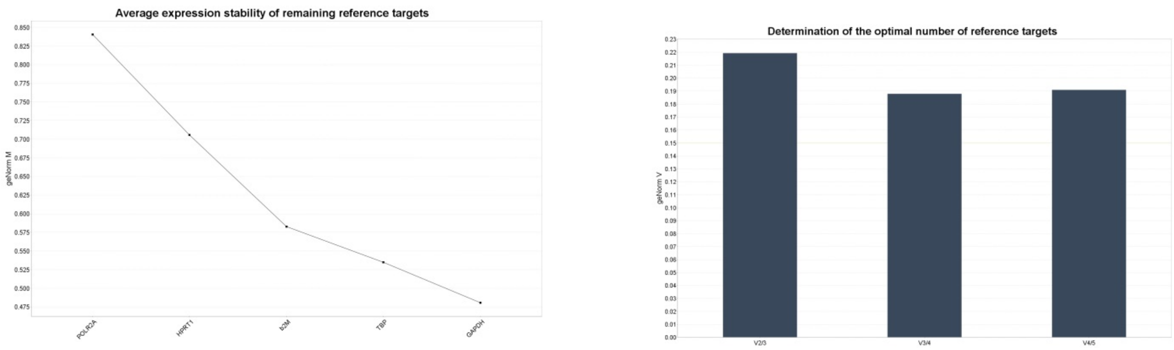

Supplementary Figure S2. geNorm M and V analysis in DCs. Before data and statistical analyses, the most stable reference genes for DCs under these stimulation conditions were calculated according to geNorm M and geNorm V values.

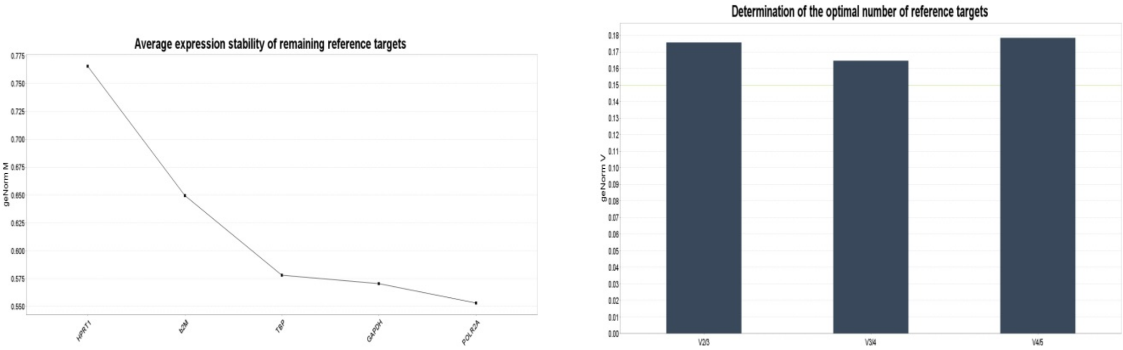

Supplementary Table S1. PDZ genes with the most significant changes in gene expression after Mϕ stimulation with HKMtb. Z-ratio value was used to represent conditions in which a particular gene had its peak changes considering the entire time course. In green are those genes that increased their expression, and in red, those that decreased. A Z-ratio > 2.0 or < –2.0 was considered significant.

Mϕ							
HKMtb							
2h		6h		12h		24h	
LIMK2	↑	APBA3	↑	AHNAK	↑	APBA3	↑
MAGI3	↑	DVL1	↑	APBA3	↑	LIMK2	↑
PDZRN4	↑	GIPC1	↑	DVL3	↑	MPP7	↑
PREX1	↑	LIMK2	↑	GIPC1	↑	PDZRN4	↑
RAPGEF2	↑	PDLIM5	↑	LIMK2	↑	PPP1R9B	↑
SIPA1	↑	PDZRN4	↑	PDLIM5	↑	PPP1R9A	↑
ARHGAP21	↓	PPP1R9B	↑	PPP1R9B	↑	RAPGEF2	↑
ARHGEF11	↓	RAPGEF2	↑	PRX	↑	SIPA1	↑
DLG4	↓	SIPA1	↑	RAPGEF2	↑	TAX1BP3	↑
DVL2	↓	ARHGAP21	↓	SCRIB	↑	ARHGAP21	↓
HTRA2	↓	ARHGEF12	↓	SIPA1	↑	DLG4	↓
LIMK1	↓	CARD11	↓	ARHGAP21	↓	DVL2	↓
LIN7B	↓	CNKSR1	↓	DLG4	↓	HTRA1	↓
MAST2	↓	DLG3	↓	DVL2	↓	IL16	↓
MAST3	↓	DLG4	↓	IL16	↓	LIMK1	↓
MPP1	↓	DVL2	↓	LIMK1	↓	LIN7B	↓
MPP5	↓	HTRA2	↓	LIN7B	↓	LIN7C	↓
MPP6	↓	LIMK1	↓	LIN7C	↓	MAST2	↓
PARD3	↓	LIN7B	↓	MAST2	↓	MAST3	↓
PDLIM2	↓	MAST2	↓	MAST3	↓	MPP1	↓
PDZRN3	↓	MAST3	↓	MPP1	↓	MPP6	↓
RGS12	↓	MPP1	↓	PARD3	↓	PDLIM2	↓

SDCBP	↓	PARD3	↓	PDLIM2	↓	PDZD4	↓
SLC9A3R2	↓	PARD6	↓	PTPN4	↓	PDZRN3	↓
		PDLIM2	↓	RGS12	↓	RGS12	↓
		PDZRN3	↓	SDCBP	↓	SDCBP	↓
		PTPN4	↓	SLC9A3R2	↓	SLC9A3R2	↓
		RGS12	↓	SNTA1	↓	SNTA1	↓
		RIMS1	↓	TIAM1	↓	TIAM1	↓
		SDCBP	↓				
		SLC9A3R1	↓				
		SLC9A3R2	↓				
		TIAM1	↓				

Supplementary Table S2. PDZ genes corresponding to the most significant changes in gene expression comparing HKMtb and LPS stimulation of Mϕ. Z-ratio value was used to represent conditions in which a particular gene had its peak changes considering the entire time course. In green are those genes that increased their expression, and in red, those that decreased. A Z-ratio > 2.0 or < –2.0 was considered significant.

Mϕ							
HKMtb				LPS			
6h		24h		6h		24h	
GIPC1	↑	MPP3	↑	CASK	↑	CYTIP	↑
MPP3	↑	MPP7	↑	CYTIP	↑	GIPC1	↑
ARHGEF12	↓	ARHGAP21	↓	DVL3	↑	GRASP	↑
PARD6	↓	IL16	↓	GIPC2	↑	LIMK2	↑
PDZRN3	↓	PDZD4	↓	GRASP	↑	PDLIM1	↑
		PDZRN3	↓	LIMK2	↑	SIPA1	↑
				MAGI3	↑	ARHGAP21	↓
				RAPGEF2	↑	DLG1	↓
				TJP1	↑	DLG5	↓
				TJP2	↑	HTRA1	↓
				CARD11	↓	IL16	↓
				CNKSR1	↓	LIMK1	↓
				DLG1	↓	LIN7A	↓
				DLG4	↓	LIN7C	↓
				DLG5	↓	MAST2	↓
				DVL2	↓	MPP1	↓
				HTRA1	↓	MPP6	↓
				HTRA2	↓	PDLIM2	↓
				IL16	↓	PDZRN3	↓
				LIN7A	↓	RAPGEF6	↓
				LIN7B	↓	RGS12	↓

MAST2	↓	SDCBP	↓
MAST3	↓	SNTA1	↓
MPP5	↓	SNTB1	↓
PDLIM2	↓	TIAM1	↓
PDZRN3	↓		
PTPN4	↓		
SIPA1L3	↓		
SLC9A3R1	↓		
SLC9A3R2	↓		
TIAM1	↓		

Supplementary Table S3. PDZ genes with the most significant changes in gene expression after the HKMtb and MC stimuli in DCs. Z-ratio value was used to represent conditions in which a particular gene had its peak changes considering the entire time course. In green are those genes that increased their expression, and in red, those that decreased. A Z-ratio > 2.0 or < –2.0 was considered significant.

DC															
HKMtb								MC							
2h		6h		12h		24h		2h		6h		12h		24h	
WHRN	↑	PPP1R9A	↑	CNKSR2	↑	CNKSR2	↑	GRASP	↑	GRASP	↑	WHRN	↑	DEPTOR	↑
DLG3	↑	CASK	↓	TJP1	↑	CYTIP	↑	CARD11	↑	CYTIP	↑	APBA3	↑	APBA3	↑
PPP1R9A	↑	DLG1	↓	DLG1	↓	DEPTOR	↑	CYTIP	↑	PARD3	↑	CYTIP	↑	CYTIP	↑
TJP1	↑	DVL2	↓	DVL2	↓	GRASP	↑	LIN7A	↑	TJP1	↑	DEPTOR	↑	ARHGEF12	↓
DLG1	↓	DVL3	↓	DVL3	↓	MPP3	↑	PDZD4	↑	AHNAK	↓	GRASP	↑	DLG1	↓
DVL2	↓	HTRA2	↓	HTRA2	↓	NOS1	↑	RAPGEF2	↑	CASK	↓	MPP3	↑	DLG5	↓
DVL3	↓	LIMK2	↓	LIMK2	↓	TJP1	↑	ARHGEF11	↓	DLG1	↓	SIPA1L1	↑	DVL2	↓
GIPC1	↓	LIN7B	↓	LIN7B	↓	DLG1	↓	DLG1	↓	DLG4	↓	TIAM1	↑	DVL3	↓
HTRA2	↓	MAGI3	↓	MAGI3	↓	DLG5	↓	DVL2	↓	DVL2	↓	TJP1	↑	ERBIN	↓
LIMK2	↓	MAST3	↓	MPDZ	↓	DVL2	↓	DVL3	↓	DVL3	↓	ARHGAP21	↓	HTRA2	↓
LIN7B	↓	MPP5	↓	MPP6	↓	DVL3	↓	HTRA2	↓	ERBIN	↓	DLG1	↓	IL16	↓
MAGI3	↓	MPP6	↓	MPP7	↓	HTRA2	↓	IL16	↓	HTRA2	↓	DVL2	↓	LIMK1	↓
MAST3	↓	MPP7	↓	PTPN4	↓	IL16	↓	LIMK2	↓	IL16	↓	DVL3	↓	LIMK2	↓
MPP6	↓	PTPN4	↓	RAPGEF6	↓	LIMK2	↓	LIN7B	↓	LIMK1	↓	ERBIN	↓	LIN7B	↓
MPP7	↓	SCRIB	↓	SIPA1	↓	LIN7B	↓	MAGI3	↓	LIMK2	↓	GIPC1	↓	MAGI3	↓
PRX	↓	SIPA1	↓	SNX27	↓	MAGI3	↓	MAST3	↓	LIN7B	↓	HTRA2	↓	MAST2	↓
PTPN4	↓	SLC9A3R1				MAST3	↓	MPP6	↓	MAGI3	↓	IL16	↓	MAST3	↓
SCRIB	↓	SNTB1				MPP5	↓	MPP7	↓	MAST3	↓	LIMK1	↓	MPP1	↓
SIPA1	↓	SNX27				MPP6	↓	PPP1R9B	↓	MPP1	↓	LIMK2	↓	MPP6	↓

SNX27	↓	MPP7	↓	PRX	↓	MPP6	↓	LIN7B	↓	MPP7	↓
		PTPN4	↓	PTPN4	↓	MPP7		MAGI3	↓	PARD6A	↓
		SIPA1	↓	SIPA1	↓	PREX1	↓	MAST3	↓	PDLIM2	↓
		SLC9A3R1	↓	SLC9A3R1	↓	PRX	↓	MPP1	↓	PTPN4	↓
		SNX27	↓	SNX27	↓	PTPN4	↓	MPP6	↓	RGS12	↓
						SIPA1	↓	MPP7	↓	SDCBP	↓
						SLC9A3R1	↓	PDLIM2	↓	SIPA1	↓
						SLC9A3R2	↓	PREX1	↓	SLC9A3R1	↓
						SNTA1	↓	PRX	↓	SNTA1	↓
						SNTB1	↓	PTPN4	↓	SNTB1	↓
						SNX27	↓	RGS12	↓	SNX27	↓
						TAX1BP3	↓	SDCBP	↓	TAX1BP3	↓
						TJP2	↓	SIPA1	↓		
								SLC9A3R1	↓		
								SNTA1	↓		
								SNTB1	↓		
								SNX27	↓		
								TAX1BP3	↓		
								TJP2	↓		

Supplementary table S4. PDZ genes with the most significant changes in gene expression in Mϕ vs DCs after the HKMtb phagocytic stimulus. Z-ratio value was used to represent conditions in which a particular gene had its peak changes considering the entire time course. In green are those genes that increased their expression, and in red, those that decreased. A Z-ratio > 2.0 or < –2.0 was considered significant.

HKMtb															
Mϕ								DC							
2h		6h		12h		24h		2h		6h		12h		24h	
DVL2	↓	APBA3	↑	APBA3	↑	APBA3	↑	PDLIM7	↑	PPP1R9A	↑	DEPTOR	↑	APBA3	↑
MAST2	↓	DVL2	↓	DVL2	↓	ARHGAP21	↓	PPP1R9A	↑	CASK	↓	PPP1R9A	↑	CYTIP	↑
		MAST2	↓	MAST2	↓	DVL2	↓	SIPA1L1	↑	DVL2	↓	TJP1	↓	DEPTOR	↑
		PARD6	↓	PDLIM2	↓	IL16	↓	ARHGEF11	↓	HTRA2	↓	DVL2	↓	GRASP	↑
		PDLIM2	↓			MAST3	↓	DLG1	↓	IL16	↓	HTRA2	↓	MPP2	↑
						PDLIM2	↓	DVL2	↓	LIN7B	↓	IL16	↓	MPP3	↑
						PDZD4	↓	HTRA2	↓	MAST3	↓	LIMK1	↓	PATJ	↑
								IL16	↓	MPP5	↓	LIN7B	↓	WHRN	↑
								LIMK1	↓	MPP6	↓	MAST3	↓	DLG1	↓
								LIN7B	↓	PTPN4	↓	MPDZ	↓	DLG4	↓
								MAST3	↓	SLC9A3R1	↓	MPP1	↓	DLG5	↓
								PREX1	↓	SNTA1	↓	MPP6	↓	DVL2	↓
								PRX	↓	SNTB1	↓	PARD6	↓	ERBIN	↓
								PTPN4	↓			PDZRN3	↓	HTRA2	↓
								SIPA1L3	↓			PTPN4	↓	IL16	↓
								SNTA1	↓			RAPGEF6	↓	LIMK1	↓
												SDCBP	↓	LIN7B	↓
												SNTA1	↓	MAST2	↓

MAST3	↓
MPP1	↓
MPP5	↓
MPP6	↓
PDLIM2	↓
PTPN4	↓
SDCBP	↓
SLC9A3R1	↓
SNTA1	↓
TJP2	↓

Supplementary Table S5. DEGs in Mϕ upon LPS stimulation. The table shows the set of DEGs of Mϕ stimulated with LPS 6 h vs unstimulated Mϕ represented in figure 4E (left column). DEGs in common between 6 and 24 h vs unstimulated Mϕ represented in figure 4D (right column).

Mϕ LPS 6h	Mϕ LPS (6h∩24h)
ARHGEF11	DLG1
CARD11	HTRA1
CYTIP	IL16
DVL2	LIMK2
DVL3	LIN7A
MAST2	PDLIM2
MAST3	RAPGEF6
MPP5	RGS12
PPP1R9B	SIPA1L1
PTPN13	SLC9A3R1
PTPN4	TIAM1
RAPGEF2	TJP1
SIPA1L3	
SLC9A3R2	
TJP2	

Supplementary Table S6. DEGs in common among time course stimulation with HKMtb in DCs. The table shows the set of 15 DEGs in common during the entire time course of DCs stimulated with HKMtb vs unstimulated DCs, represented in Figure 5C.

HKMtb (2h ∩6h∩12h∩24h)
DLG1
DVL2
IL16
LIMK2
LIN7B
MAGI3
MAST3
MPP1
MPP6
MPP7
PTPN4
SIPA1
SLC9A3R1
SNTB1
SNX27

Supplementary Table S7. DEGs overlapped upon time course stimulation with MC in DCs. The table shows the set of 18 DEGs in common during the entire time course of DCs stimulated with MC vs unstimulated DCs (right column). The five DEGs at 12 of stimulation (middle column) and the four DEGs in common at 12 and 24 of MC stimulation (right column). All these DEGs are represented in Figure 5D.

MC (2h ∩ 6h ∩ 12h ∩ 24h)	MC 12h	MC (12h ∩ 24h)
DLG1	APBA3	ARHGAP21
DVL3	CYTIP	MAST2
HTRA2	PPP1R9A	PATJ
IL16	PTPN13	SDCBP
LIMK1	SNTA1	
LIMK2	TIAM1	
LIN7B		
MAGI3		
MAST3		
MPP1		
MPP7		
PDLIM2		
PRX		
PTPN4		
SIPA1		
SLC9A3R1		
SNTB1		
SNX27		

Supplementary Table S8. The human PDZ genes. The 153 human PDZ genes reported in HUGO database are listed. Aliases and accession numbers are included. (modified from: HUGO Gene Nomenclature Committee at the European Bioinformatics Institute; https://www.genenames.org/data/genegroup/#!/group/1220)

HGNC ID (gene)	Approved symbol	Approved name	Previous symbols	Aliases
HGNC:347	AHNAK	AHNAK nucleoprotein		MGC5395,AHNAK1
HGNC:20125	AHNAK2	AHNAK nucleoprotein 2	C14orf78	
HGNC:578	APBA1	amyloid beta precursor protein binding family A member 1	MINT1	D9S411E,X11
HGNC:579	APBA2	amyloid beta precursor protein binding family A member 2	X11L,MINT2	D15S1518E,LIN-10,MGC:14091,HsT16821
HGNC:580	APBA3	amyloid beta precursor protein binding family A member 3		X11L2,mint3
HGNC:23725	ARHGAP21	Rho GTPase activating protein 21		KIAA1424,ARHGAP10
HGNC:29293	ARHGAP23	Rho GTPase activating protein 23		KIAA1501
HGNC:14580	ARHGEF11	Rho guanine nucleotide exchange factor 11		KIAA0380,GTRAP48,PDZ-RHOGEF
HGNC:14193	ARHGEF12	Rho guanine nucleotide exchange factor 12		KIAA0382,LARG
HGNC:16422	CARD10	caspase recruitment domain family member 10		CARMA3, BIMP1
HGNC:16393	CARD11	caspase recruitment domain family member 11		CARMA1,BIMP3
HGNC:16446	CARD14	caspase recruitment domain family member 14	PSORS2	CARMA2,BIMP2
HGNC:1497	CASK	calcium/calmodulin dependent serine protein kinase	TNRC8	LIN2,CAGH39,FGS4
HGNC:19700	CNKSR1	connector enhancer of kinase suppressor of Ras 1		CNK1,KSR,CNK

HGNC:19701	CNKSR2	connector enhancer of kinase suppressor of Ras 2		KIAA0902,CNK2,KSR2
HGNC:23034	CNKSR3	CNKSR family member 3	MAGI1	FLJ31349
HGNC:9506	CYTIP	cytohesin 1 interacting protein	PSCDBP	B3-1,HE,CYBR,CASP,CYTHIP
HGNC:22953	DEPTOR	DEP domain containing MTOR interacting protein	DEPDC6	DEP.6,FLJ12428
HGNC:2900	DLG1	discs large MAGUK scaffold protein 1		SAP97,SAP-97,hdlg,DLGH1,dJ1061C18.1.1
HGNC:2901	DLG2	discs large MAGUK scaffold protein 2		PSD-93,PSD93,chapsyn-110,PPP1R58
HGNC:2902	DLG3	discs large MAGUK scaffold protein 3		NE-Dlg,SAP102,SAP-102,NEDLG,KIAA1232,MRX90,PPP1R82
HGNC:2903	DLG4	discs large MAGUK scaffold protein 4		PSD-95,PSD95,SAP90,SAP-90
HGNC:2904	DLG5	discs large MAGUK scaffold protein 5		P-dlg,KIAA0583
HGNC:3084	DVL1	dishevelled segment polarity protein 1		
HGNC:3086	DVL2	dishevelled segment polarity protein 2		
HGNC:3087	DVL3	dishevelled segment polarity protein 3		KIAA0208
HGNC:15842	ERBIN	erbb2 interacting protein	ERBB2IP	LAP2
HGNC:29159	FRMPD1	FERM and PDZ domain containing 1		KIAA0967,FRMD2
HGNC:29382	FRMPD3	FERM and PDZ domain containing 3		RP5-1070B1.1,KIAA1817
HGNC:1226	GIPC1	GIPC PDZ domain containing family member 1	C19orf3,RGS19IP1	TIP-2,Hs.6454,GIPC,SEMCAP,GLUT1CBP,SYNECTIN,NIP
HGNC:18177	GIPC2	GIPC PDZ domain containing family member 2		FLJ20075,SEMCAP-2

HGNC:18183	GIPC3	GIPC PDZ domain containing family member 3	C19orf64,DFNB72,DFNB15	DFNB95
HGNC:17643	GOPC	golgi associated PDZ and coiled-coil motif containing		dJ94G16.2,PIST,FIG,GOPC1,CAL
HGNC:16769	GORASP1	golgi reassembly stacking protein 1	GOLPH5	GRASP65,P65,FLJ23443
HGNC:17500	GORASP2	golgi reassembly stacking protein 2		GRASP55,GRS2,GOLPH6
HGNC:18707	TAMALIN	trafficking regulator and scaffold protein tamalin	GRASP	
HGNC:18464	GRID2IP	Grid2 interacting protein		
HGNC:18708	GRIP1	glutamate receptor interacting protein 1		
HGNC:23841	GRIP2	glutamate receptor interacting protein 2		KIAA1719
HGNC:9476	HTRA1	HtrA serine peptidase 1	PRSS11	HtrA,IGFBP5-protease,ARMD7
HGNC:14348	HTRA2	HtrA serine peptidase 2	PRSS25	OMI,PARK13
HGNC:30406	HTRA3	HtrA serine peptidase 3		Tasp,Prsp
HGNC:26909	HTRA4	HtrA serine peptidase 4		FLJ90724
HGNC:5980	IL16	interleukin 16		LCF,IL-16,prIL-16,HsT19289,FLJ42735,FLJ16806
HGNC:28881	PATJ	PATJ crumbs cell polarity complex component	INADL	Cipp
HGNC:6613	LIMK1	LIM domain kinase 1		LIMK
HGNC:6614	LIMK2	LIM domain kinase 2		
HGNC:17787	LIN7A	lin-7 homolog A, crumbs cell polarity complex component		MALS-1,TIP-33,LIN-7A,VELI1

HGNC:17788	LIN7B	lin-7 homolog B, crumbs cell polarity complex component		MALS-2,LIN-7B,VELI2
HGNC:17789	LIN7C	lin-7 homolog C, crumbs cell polarity complex component		MALS-3,LIN-7C,LIN-7-C,VELI3,FLJ11215
HGNC:6646	LMO7	LIM domain 7	FBXO20	FBX20,KIAA0858
HGNC:18531	LRRC7	leucine rich repeat containing 7		KIAA1365,densin-180
HGNC:30006	MAGIX	MAGI family member, X-linked		PDZX,JM10,FLJ21687
HGNC:946	MAGI1	membrane associated guanylate kinase, WW and PDZ domain containing 1	BAIAP1	BAP1,MAGI-1,TNRC19,AIP3,WWP3
HGNC:18957	MAGI2	membrane associated guanylate kinase, WW and PDZ domain containing 2		AIP1,ARIP1,KIAA0705,ACVRIP1,MAGI-2
HGNC:29647	MAGI3	membrane associated guanylate kinase, WW and PDZ domain containing 3		MAGI-3
HGNC:19034	MAST1	microtubule associated serine/threonine kinase 1		SAST,KIAA0973
HGNC:19035	MAST2	microtubule associated serine/threonine kinase 2		MAST205,KIAA0807
HGNC:19036	MAST3	microtubule associated serine/threonine kinase 3		KIAA0561
HGNC:19037	MAST4	microtubule associated serine/threonine kinase family member 4		KIAA0303
HGNC:7137	AFDN	afadin, adherens junction formation factor	MLLT4	AF-6,AF6
HGNC:7208	MPDZ	multiple PDZ domain crumbs cell polarity complex component		MUPP1
HGNC:7219	MPP1	MAGUK p55 scaffold protein 1	DXS552E	PEMP
HGNC:7220	MPP2	MAGUK p55 scaffold protein 2	DLG2	DKFZp761D0712
HGNC:7221	MPP3	MAGUK p55 scaffold protein 3	DLG3	

HGNC:13680	MPP4	MAGUK p55 scaffold protein 4	DLG6	
HGNC:18669	PALS1	protein associated with LIN7 1, MAGUK p55 family member	MPP5	FLJ12615
HGNC:18167	PALS2	protein associated with LIN7 2, MAGUK p55 family member	MPP6	VAM-1,p55T
HGNC:26542	MPP7	MAGUK p55 scaffold protein 7		FLJ32798
HGNC:31104	MYO18A	myosin XVIIIA	TIAF1	KIAA0216,MysPDZ
HGNC:7872	NOS1	nitric oxide synthase 1	NOS	nNOS
HGNC:16051	PARD3	par-3 family cell polarity regulator		PAR3,PARD3A,Bazooka,Baz,ASIP,PPP1R118
HGNC:14446	PARD3B	par-3 family cell polarity regulator beta	ALS2CR19	Par3L,PAR3beta
HGNC:15943	PARD6A	par-6 family cell polarity regulator alpha		PAR-6,PAR-6A,TAX40,PAR6alpha,TIP-40
HGNC:16245	PARD6B	par-6 family cell polarity regulator beta		PAR-6B
HGNC:16076	PARD6G	par-6 family cell polarity regulator gamma		PAR-6G,PAR6gamma
HGNC:13406	PCLO	piccolo presynaptic cytomatrix protein		KIAA0559,DKFZp779G1236,ACZ
HGNC:2067	PDLIM1	PDZ and LIM domain 1	CLIM1	CLP-36,hCLIM1,CLP36
HGNC:13992	PDLIM2	PDZ and LIM domain 2		
HGNC:20767	PDLIM3	PDZ and LIM domain 3		ALP
HGNC:16501	PDLIM4	PDZ and LIM domain 4		RIL
HGNC:17468	PDLIM5	PDZ and LIM domain 5		LIM,Enh

HGNC:15710	LDB3	LIM domain binding 3	CMD1C	PDLIM6,KIAA0613,ZASP
HGNC:22958	PDLIM7	PDZ and LIM domain 7		ENIGMA
HGNC:8821	PDZK1	PDZ domain containing 1		PDZD1,NHERF3
HGNC:18486	PDZD2	PDZ domain containing 2	PDZK3	KIAA0300
HGNC:19891	PDZD3	PDZ domain containing 3	PDZK2	FLJ22756,IKEPP,NHERF4
HGNC:21167	PDZD4	PDZ domain containing 4	PDZK4	KIAA1444,LU1,FLJ34125,PDZRN4L,LNX5
HGNC:16843	FRMPD2B	FERM and PDZ domain containing 2B, pseudogene	PDZK5A,PDZD5A,FRMPD2L1,FRMPD2P2,PDZK5B,PDZD5B,FRMPD2L2,FRMPD2P1	yX59F3.2,bA556L1.2
HGNC:28572	FRMPD2	FERM and PDZ domain containing 2	PDZD5C,PDZK5C	MGC35285
HGNC:29239	INTU	inturned planar cell polarity protein	PDZK6,PDZD6	KIAA1284,CPLANE4
HGNC:26257	PDZD7	PDZ domain containing 7	PDZK7,DFNB57	FLJ23209,bA108L7.8
HGNC:12597	USH1C	USH1 protein network component harmonin	DFNB18	PDZ73,harmonin,NY-CO-37,NY-CO-38,PDZ-73,AIE-75,PDZD7C
HGNC:26974	PDZD8	PDZ domain containing 8	PDZK8	bA129M16.2,FLJ34427
HGNC:28740	PDZD9	PDZ domain containing 9	C16orf65	MGC50721
HGNC:29007	FRMPD4	FERM and PDZ domain containing 4	PDZK10,PDZD10	KIAA0316
HGNC:28034	PDZD11	PDZ domain containing 11	PDZK11	
HGNC:16854	RAPGEF2	Rap guanine nucleotide exchange factor 2	PDZGEF1	PDZ-GEF1,RA-GEF,DKFZP586O1422,KIAA0313
HGNC:20655	RAPGEF6	Rap guanine nucleotide exchange factor 6	PDZGEF2	RA-GEF-2,PDZ-GEF2

HGNC:51488	PDZPH1P	PDZ and pleckstrin homology domains 1, pseudogene		
HGNC:20421	LNX2	ligand of numb-protein X 2	PDZRN1	MGC46315
HGNC:6657	LNX1	ligand of numb-protein X 1	LNX	MPDZ,PDZRN2
HGNC:17704	PDZRN3	PDZ domain containing ring finger 3		KIAA1095,SEMACAP3,LNX3,SEMCAP3
HGNC:30552	PDZRN4	PDZ domain containing ring finger 4		DKFZp434B0417,LNX4,FLJ33777,IMAGE5767589
HGNC:16361	WHRN	whirlin	DFNB31	CIP98,USH2D,PDZD7B
HGNC:9394	PICK1	protein interacting with PRKCA 1	PRKCABP	dJ1039K5,MGC15204
HGNC:14946	PPP1R9A	protein phosphatase 1 regulatory subunit 9A		Neurabin-I,KIAA1222,FLJ20068
HGNC:9298	PPP1R9B	protein phosphatase 1 regulatory subunit 9B	PPP1R6,PPP1R9	Spn,SPINO
HGNC:32594	PREX1	phosphatidylinositol-3,4,5-trisphosphate dependent Rac exchange factor 1		KIAA1415,P-REX1
HGNC:22950	PREX2	phosphatidylinositol-3,4,5-trisphosphate dependent Rac exchange factor 2	DEPDC2	DEP.2,FLJ12987,P-REX2,PPP1R129
HGNC:13797	PRX	periaxin		KIAA1620
HGNC:9567	PSMD9	proteasome 26S subunit, non-ATPase 9		p27,Rpn4
HGNC:9655	PTPN3	protein tyrosine phosphatase non-receptor type 3		PTPH1
HGNC:9656	PTPN4	protein tyrosine phosphatase non-receptor type 4		PTPMEG
HGNC:9646	PTPN13	protein tyrosine phosphatase non-receptor type 13		PTP1E,PTP-BAS,PTPL1,PTP-BL
HGNC:22226	RADIL	Rap associating with DIL domain		FLJ10324,KIAA1849,RASIP2

HGNC:9999	RGS3	regulator of G protein signaling 3		C2PA,FLJ20370,PDZ-RGS3
HGNC:9994	RGS12	regulator of G protein signaling 12		
HGNC:19973	RHPN1	rhophilin Rho GTPase binding protein 1		KIAA1929,RHPN,ODF5
HGNC:19974	RHPN2	rhophilin Rho GTPase binding protein 2		
HGNC:17282	RIMS1	regulating synaptic membrane exocytosis 1	RAB3IP2,CORD7	RIM,KIAA0340,RIM1
HGNC:17283	RIMS2	regulating synaptic membrane exocytosis 2	RAB3IP3	KIAA0751,RIM2,OBOE
HGNC:30377	SCRIB	scribble planar cell polarity protein		KIAA0147,SCRB1,Vartul
HGNC:10662	SDCBP	syndecan binding protein		SYCL,MDA-9
HGNC:15756	SDCBP2	syndecan binding protein 2		ST-2,SITAC18
HGNC:15474	SHANK1	SH3 and multiple ankyrin repeat domains 1		SSTRIP,SPANK-1,synamon
HGNC:14295	SHANK2	SH3 and multiple ankyrin repeat domains 2	CORTBP1	CTTNBP1,ProSAP1,SHANK,SPANK-3
HGNC:14294	SHANK3	SH3 and multiple ankyrin repeat domains 3		SPANK-2,prosap2,KIAA1650,PSAP2
HGNC:630	SHROOM2	shroom family member 2	APXL	
HGNC:30422	SHROOM3	shroom family member 3		ShrmL,SHRM,KIAA1481,APXL3
HGNC:29215	SHROOM4	shroom family member 4		KIAA1202
HGNC:10885	SIPA1	signal-induced proliferation-associated 1		SPA1
HGNC:20284	SIPA1L1	signal induced proliferation associated 1 like 1		KIAA0440,E6TP1,SPAR1

HGNC:23800	SIPA1L2	signal induced proliferation associated 1 like 2		KIAA1389,SPAR2
HGNC:23801	SIPA1L3	signal induced proliferation associated 1 like 3		KIAA0545,SPAR3
HGNC:11075	SLC9A3R1	SLC9A3 regulator 1		NHERF,EBP50,NHERF1
HGNC:11076	SLC9A3R2	SLC9A3 regulator 2		SIP-1,TKA-1,NHERF-2,E3KARP
HGNC:11167	SNTA1	syntrophin alpha 1	SNT1	TACIP1,LQT12
HGNC:11168	SNTB1	syntrophin beta 1	SNT2B1	59-DAP,A1B,BSYN2,TIP-43,SNT2
HGNC:11169	SNTB2	syntrophin beta 2	SNT2B2,SNTL,D16S2531E	EST25263,SNT3
HGNC:13740	SNTG1	syntrophin gamma 1		SYN4,G1SYN
HGNC:13741	SNTG2	syntrophin gamma 2		SYN5,G2SYN
HGNC:20073	SNX27	sorting nexin 27		MY014,KIAA0488,MGC20471
HGNC:19694	STXBP4	syntaxin binding protein 4		Synip,MGC50337
HGNC:18955	SYNJ2BP	synaptojanin 2 binding protein		Arip2
HGNC:17732	SYNPO2	synaptopodin 2		MYOPODIN,SYISL
HGNC:23532	SYNPO2L	synaptopodin 2 like		FLJ12921
HGNC:30684	TAX1BP3	Tax1 binding protein 3		TIP-1
HGNC:11805	TIAM1	TIAM Rac1 associated GEF 1		
HGNC:11806	TIAM2	TIAM Rac1 associated GEF 2		STEF

HGNC:11827	TJP1	tight junction protein 1		ZO-1,MGC133289,DKFZp686M05161
HGNC:11828	TJP2	tight junction protein 2	DFNA51	ZO-2,X104,ZO2
HGNC:11829	TJP3	tight junction protein 3		ZO-3

Supplementary Table S9. Pair of primers used

Target	FP 5′->3′	RP 5′->3′
AHNAK	GCAAGGCATTCGCTCCTGA	AGCCACTACCCTGCCAGTT
APBA3	CTACAAGATGCTCTGCCACGTA	TCCCGTAGGAACTGGCTGTA
ARHGAP21	GGAAGGGTGGCTTCATTTCC	TCCATGGCCGAATACTTCCA
ARHGEF11	GTGGCTACCGTTCTTCCCTA	GTTTCGCTTCTTGTCCTCCAA
ARHGEF12	TTGATTTCCCACCACCTCCA	TGGTGTCCCATGTTCAGTCA
b2M	TTAGCTGTGCTCGCGCTAC	CTCTGCTGGATGACGTGAGTAA
CARD10	CACAGCTCAGTGAGGAGAAGAA	CACTTTGAGCTTGAGCTGATCC
CARD11	GCTGCCATCCAAGATCAACC	TCTCCAAGAAGACCACATAGCC
CARD14	CCTGGATTATGAGCTCCTAGACAC	CCGAGACATCAAGCCTTCCA
CASK	GCTGACGCTGGTTTTGTGTA	CAGTAGCGTAGAGCTTCCAGTA
CNKSR1	CTTCATCTTCGCTGCTGATAC	GACTGGTACTTGGAGATGCA
CNKSR2	TGCTGACTGCAGGATATGCA	CTTCCTTGTCACTCTCACTCCA
CYTIP	CCTGATCAGATCGTCCGGAAA	GCTTCAAGCTCCGTTCTTTTCA
DEPTOR	AGTAAGCCATGCCACATCCA	TGACAGAGACGACAAACTGACA
DLG1	AACTAGCCAGAAGCGATCCC	CTGGGAAGCCCACTGTCTTTA
DLG2	GGATCCCTGCGAACCAATCA	CCTTGCTCTTGTCGTAGTCGAA
DLG3	GATCGGTGTGATCCCCAGTAA	GTCCTGGCATGGAACTTCAC
DLG4	AGCTGGAGCAGGAGTTCAC	ACACGCTTCACCTTGTGGTA
DLG5	CGGATTCCATGGAGTGGGAAA	TCAAACCCCAGTGCCTTCAA
DVL1	CAAGAACGTGCTCAGCAACC	GGAAGCTTGGCATTGTCATCAAA
DVL2	TGCCTCCCGCCTCCTTAA	TGACGCTGCTGAAGGATGAC
DVL3	TTTGCAGCGACCCAGCTATA	CATCCGAGATCTCCTCCTTCAC
ERBIN	TTCCCTCAACAGCCAAGGAC	GTTTCCTCTGTTCCTCCCACAA
GAPDH	GAACGGGAAGCTTGTCATCAA	ATCGCCCCACTTGATTTTGG
GIPC1	CTGGCTCTGGCCCACAA	GCAGGTCATCCACCTTCTCAA
GIPC2	CTGAGGATTCACTTGGTCTCA	TCCCCAACACAGATTGTTTTAAC
GRASP	ACCTTTGTCTGCCGAGTTCA	CTTCCACATTCAGGCCATTGAC
HPRT1	GCTTTCCTTGGTCAGGCAGTA	ACTTCGTGGGGTCCTTTTCAC
HTRA1	CGGGAGGCCCGTTAGTAAA	CAAAGGAGATTCCAGCTGTCAC
HTRA2	TCTGGAGGTCCCCTGGTTAA	GAGATTCCAGCTGTGACCTTCA

IL16	GCAGCCAGTGATGTTTCTGTA	CCTGCCGACATCTTCTCCA
LIMK1	TGAGAAGGTGGATGTGTTCTCC	GTAGTCAGGGTCTGCGTTCA
LIMK2	TGAGACGGTGGATATCTTCTCC	AGGCAGTCAGGATCTGCATA
LIN7A	AGACCAGCTGCTATCAGTGAA	AGCAGCCTTGAGTAGTTCCA
LIN7B	AAGCTGGTTGTCCGTTACACA	GGTTTCAACCTCGAGACTCCAA
LIN7C	GGACATCAGTAGCAGTCCTGAA	TGTCCTTCACTGGCAGCAA
MAGI1	GTTCAAAGCACCCCAAGCAA	CTCGAAGGCTAAAGCCAAATCC
MAGI2	TTCAGCTGCAAGGACACGAA	GCTGTCGGATGTCTGGTTTCA
MAGI3	GTCCTCCTTCACCAACCAAA	CATTTATGGCCTCCAAACTTCC
MAST2	TCCAAACTGCTCCACCAGAA	AAAGAATGGGTGCTGCTTCAC
MAST3	TTCCGGCTATGGAACCAACA	AAGGGAAGCTGGTGGAGAC
MPDZ	GGGCTGACGTCAAGCAGTATA	TCTGGTCCTCGCTCTAGTGTA
MPP1	CCTGCATTCAAGAGGAAGACC	GCTGAGCAGGGCATTCTTAA
MPP2	GGCAGCCTTTCAGGAAAGAA	GCAGCTCATGACGGTCAAA
MPP3	GTCCGCTTGGTGAAGAACAA	TGATCCTGGCCACCACAA
MPP4	AGGGACTGGACCCTGAACA	AGAGACTGGAACCACCTTGAAC
MPP5	GGGAAGGGGACGAAGATAATCA	GCTTCCCTTTGCTGCTGAAA
MPP6	TCACAAAAGAGCTGGGGAAC	CATGGAGGATTCGGGCAATTA
MPP7	ACCGTATCGGCGACAAACTA	TCAGTTCATTCAGCCCTACTCC
NOS1	TTCCACCAGGAGATGCTCAAC	TTCCAGACATGCGTGTTCCA
PARD3	GAACCACGCAGATTTGGGAA	TTCACCCGAAGCCTTCCA
PARD6A	GCGCCTACTGGTGCAGAA	CTGCAGAGAGTTGGAGGCAAA
PATJ	TCCCTTTCCAGTGCCATCAA	GCTGCCATCTTCCTCACTACTA
PDLIM1	TCAACAATGCCCTGGAGTCAAA	TGGAGGCTGAGCATGGTCTA
PDLIM2	GATTTCCACACGCCCATCA	GATGGCCACGATTATGTCTCC
PDLIM5	CACCGCGAGTCACTTGTCA	GGGCCAACCAGTGACACA
PDLIM7	AGGCCAGTCGCGTTCCTT	TGCTCCTCATCCGGGTCTTG
PDZD4	ACAGCATTGCAGCCAAAGAC	GTTCTGGACGTCTACACCGTTA
PDZRN3	GGCCATGCAATTCACAGCTA	TGGACAAGATGGTGGCTGTA
PDZRN4	CCTGCTGATGCAGACAGAAC	CCCAGCTTCTCTTGACTGCTA
POLR2A	CTCGCCTCTTCTACTCCAACA	ATGGAGTCCCCAATGCCAATA
PPP1R9A	AGAAGGTGGTGCTGCTCAA	ACCCACCAAGCTGATTCCA
PPP1R9B	CGAGCCGGAAGATCCATTTCA	TCGTTGCGACGATCGTAATCC
PREX1	TACGACGATGGCACCTACAA	AGACGGCAGTAAAGCCTCA
PRX	CGGCGGCAAAGAGGGAA	GAAGAACACTCGGGCACTCA
PTPN13	TGAGGGCAATGACCCTTGAA	TCTGGCCAGGCAGTGAAA
PTPN4	TGCTGCCCTTTTAGCTTCA	TCTGAGAGGTAGCCTGACAA
RAPGEF2	GAACTGGAACAAGAAAGGGACAC	GAATGCTCTTCCACCAAATGCA
RAPGEF6	GTTGTAGCATTGCCAGTGAC	ACATCTCCTACCTCACTCTCC
RGS12	AAGGGACCTGCGCCTAGAAA	TGAGTCCCACTGACCGGTTAA
RIMS1	TCACACAAAAGCCTGGTTCC	GCTATACAGGCCCCATTTTCC

SCRIB	AAGACGCCGAAGAGGACTA	TCGATCTCCCTGTCATCCC
SDCBP	TGTCTCTCTATCCATCTCTCGAA	TGGCAGGGTTTGCAGAAA
SHANK3	GCAGTTTGCAAAGCTTCACAC	ACCTTGTCCGTGCTATGCA
SIPA1	ATGCTGCCTTACACCCCTAA	CTGGAACACGATGGTCACAA
SIPA1L1	TGTCACGGTGAAGGTTGTCA	TGCGGTAGGTTTCAGAGCAA
SIPA1L3	TACGTGAGATACAAGCCATCCC	GTGGCTGAAGTGGGGATCA
SLC9A3R1	GGGGCTGGCAACGAAAA	GGGGCCCTTCTTCATGGTA
SLC9A3R2	TCACCCGTCACCAATGGAA	AGTGTCCTTGTCGGAACCA
SNTA1	TGCAGGTCCTCAAGAAGACA	AATACGGTGAGACGTCCTTCA
SNTB1	CCTGGTTCAGCCCAGTTCA	CCTGGGGTGATCCCTTTCC
SNX27	GACGTCGGGGATTGGAAGAATA	TCCTGCATGATGTCACTCTCAC
TAX1BP3	GAGCAGGGTCGAGATGTCCTA	ACCTTGACGCAGCTTGTGAA
TBP	TGCCCGAAACGCCGAATATA	CGTGGTTCGTGGCTCTCTTA
TIAM1	ACTGCTGTGGTCCTTGTGTA	AAATGGAAAGCCTGTGAGATCC
TJP1	AGCCAGCCTGCTAAACCTA	TGGCTTGCCAATCGAAGAC
TJP2	CACTGGAGTGGTCCGGTTAAA	GCTTTCGGAGTCACATCCAGTA
WHRN	TCGTCATGGCCCTGTTCAA	GGGGAAATGGTGCCTCTCA
